# Slow-Breathing Curriculum for Stress Reduction in High School Students: Lessons Learned From a Feasibility Pilot

**DOI:** 10.3389/fresc.2022.864079

**Published:** 2022-07-01

**Authors:** Tanya G. K. Bentley, Cerena Seeber, Emily Hightower, Brian Mackenzie, Rob Wilson, Aly Velazquez, Anna Cheng, Nicholas N. Arce, Kent A. Lorenz

**Affiliations:** ^1^The Health and Human Performance Foundation, Los Angeles, CA, United States; ^2^Aspen High School, Aspen, CO, United States; ^3^Department of Kinesiology, San Francisco State University, San Francisco, CA, United States

**Keywords:** diaphragmatic breathing, feasibility pilot, stress reduction, adolescents, high school

## Abstract

**Purpose:**

Nearly one in three US adolescents meet the criteria for anxiety, an issue that has worsened with the COVID-19 pandemic. We developed a video-based slow diaphragmatic breathing stress-reduction curriculum for high school students and evaluated its feasibility, tolerability, and preliminary effectiveness.

**Methods:**

This cluster-randomized feasibility pilot compared 5-min slow diaphragmatic breathing for 5 weeks with treatment-as-usual control among four 12th-grade public high school classes. Students individually participated after school during COVID-19-related hybrid teaching, with slow diaphragmatic breathing three times/week and breath science education once/week. Feasibility was based on completion of breathing exercises, breath science education, and preliminary effectiveness assessments, and ease/tolerability was based on qualitative assessments. Preliminary effectiveness was measured with the State-Trait Anxiety Inventory (STAI) and a timed-exhale carbon dioxide tolerance test (CO_2_TT) of physiological stress response. Descriptive statistics and repeated analysis of variance were performed to quantify and compare outcomes between time periods. Human subjects research approval was granted through Western IRB–Copernicus Group (WCG IRB) [ClinicalTrials.gov, Identifier: NCT05266833.]

**Results:**

Forty-three students consented to participate. Breath practice compliance ranged from 29 to 83% across classes and weeks, and decreased on average over the 5 weeks. Compliance with the breath science videos ranged from 43 to 86%, and that with the weekly STAI-State and CO_2_TT measures varied from 36 to 86%. Compliance with ease/tolerability assessments ranged from 0 to 60%. Preliminary effectiveness assessments' compliance varied across classes from 83 to 89% during baseline, and 29 to 72% at follow-up. The curriculum was rated as somewhat-to-definitely useful/beneficial, and definitely-to-very easy/tolerable. Students reported enjoying the diaphragmatic breathing, CO_2_TT, and breath science education; some found the extended exhales challenging and the curriculum and assessments time-consuming. Preliminary effectiveness analyses indicated no significant changes in STAI or CO_2_TT from baseline to followup or from before to after breathing exercises (*p* > 0.05 for all).

**Conclusions:**

Implementation of this 5-week slow breathing curriculum was feasible and tolerable to this cohort. Compliance, tolerability, and effectiveness may be improved with in-class participation. Future research on simple and accessible slow-breathing exercises is warranted to address today's adolescent stress-management crisis.

**Trial Registration:**

ClinicalTrials.gov, Identifier: NCT05266833

## Introduction

Stress during adolescence is an all-too common phenomenon. In 2013, over 30% of US adolescents reported feeling overwhelmed, depressed, or sad due to stress, and nearly one in three American adolescents in 2010 met the criteria for anxiety (1, 2). Sources of unmanageable stress during adolescence are wide-ranging, including rapid socioemotional changes, social pressures, school demands, and safety or financial concerns (3–5). Psychological wellbeing among adolescents has worsened in recent years, and challenges for today's youth associated with the COVID-19 pandemic have only added to these stresses (6, 7). Untreated adolescent stress and anxiety can adversely affect teenagers' development, educational attainments, and physical and mental health, increasing likelihood of suicide, substance abuse, disordered eating, and sexual risk-taking (2, 8, 9).

The World Health Organization encourages adolescents to develop skills for managing emotions, but nearly half of teens report they are not doing enough or are not sure if they are doing enough to manage their stress (2, 8). Diaphragmatic breathing is recommended by the American Academy of Child and Adolescent Psychiatry as one adolescent stress management strategy (3). Diaphragmatic breathing involves intentional slow breaths that start from the diaphragm or abdominal area and focus on abdominal, then lung, then chest expansion during the inhale and a slow, gradual, full release of air on the exhale. These practices improve psychological and physiological stress responses (10–16), and in youth and adolescents, have been shown to significantly reduce in-the-moment “state” and general “trait” anxiety, sports-related anxiety, and test-related anxiety (17–24).

Most research indicates that these benefits come about from slow diaphragmatic breathing's impact on rebalancing the autonomic nervous system and stimulating the vagus nerve (14, 25, 26). These effects downregulate the sympathetic nervous system's “fight or flight” response and increase activation of the parasympathetic nervous system's “recover and repair” response. This improves the body's natural homeostasis and innate stress-recovery capacities, and regulates cortisol release (27). By allowing the breath to access the lower and often unused portions of the lungs, diaphragmatic breathing also increases the body's oxygen (O_2_) absorption and its tolerance to endogenous CO_2_, a marker of physiological stress response (27–31). The practice is considered safe and potentially suitable for virtually any population (11, 19, 25, 32, 33).

The US Centers for Disease Control and Prevention recommend that schools play a role in providing support for reducing adolescent stress (34). Diaphragmatic breathing provides a simple, no-cost, scalable solution and may be an effective school-based intervention. Although several school-based programs exist that incorporate mindful breathing, a mindfulness-based approach to bringing awareness to one's natural breathing patterns (35, 36), research on intentional, voluntary diaphragmatic breathing practices in this setting is lacking. These practices differ from mindful breathing in their application of controlled breath-pattern regulation that deliberately creates a slower-paced and deeper breath cycle. The addition of this technique in the high school setting, if proven feasible, would provide teens with a simple, scalable and accessible tool to add to their stress-management toolkit.

We developed a video-based, 5-week, slow diaphragmatic breathing stress-reduction curriculum for high school students and conducted a cluster-randomized pilot study to examine its feasibility and tolerability. To provide preliminary insights into the potential benefits of such an intervention and the feasibility of implementing a larger effectiveness study, we sought as a secondary aim to describe changes in participants' short- and long-term stress levels. We used the lessons learned here to provide recommendations for the design of future interventions such as this for stress-reduction in high school settings.

## Materials and Methods

### Participants

**Figure 1:**
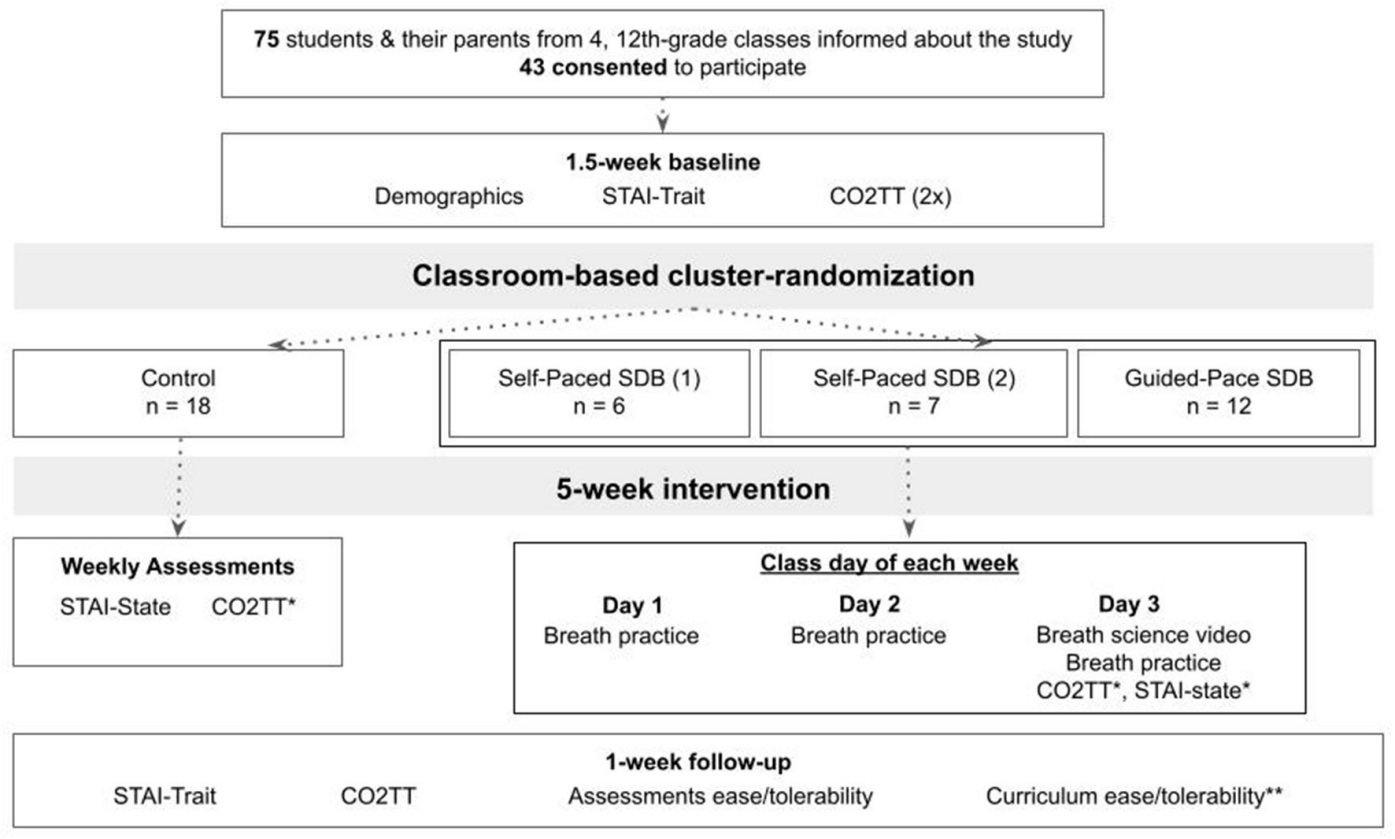
Participant flow through study recruitment, procedures, data collection, and intervention. CO_2_TT, carbon dioxide tolerance test; SDB, slow diaphragmatic breathing; STAI, State-Trait Anxiety Inventory. *For the control class, CO_2_TT was done each week once before and once after STAI-State; for the breathing classes, CO_2_TT and STAI-State were both done each week once before and once after that day's breathing practices and the breath science video. **Only for participants in the breathing classes.

This cluster-randomized pilot study compared 5-min slow diaphragmatic breathing with treatment-as-usual among high school students. Students were recruited from a public high school in Colorado, United States, where a teacher identified through the study investigators had expressed interest in implementing the curriculum in her classes. The only inclusion criterion applied was being a student in one of four English classes taught by this teacher, and there were no further exclusion criteria. Students were informed about the study during class, and their parents were notified via email. The study procedures were fully explained to the participants and their parents during the recruitment process, and participants were told they would not be compensated for participating in the study. Forty-three students and their parents signed informed written assent (students) and consent (parents) to participate in the study (Figure 1). Signatures were provided outside of school time via Research Electronic Data Capture (REDCap) tools, a secure online repository system hosted at Vanderbilt University (37, 38). Human subjects research approval was granted through Western IRB–Copernicus Group (WCG-IRB).

### Procedures

All study procedures were completed by participants individually after school during COVID-19-related hybrid instruction. Study components, including breath practice and breath science education videos, were emailed to participants through REDCap and completed in this platform on participants' individual computers or electronic tablets.

The study randomization, data collection, and intervention processes are outlined in Figure 1. During the 1.5-week baseline period following consent, participants completed baseline demographics and psychometric and physiologic stress assessments (Supplementary Appendices 1–3). Cluster-randomization by class was done immediately following completion of all baseline assessments, with the classes randomly assigned to 2 breathing curricula, self-paced or guided-pace slow diaphragmatic breathing (SP and GP, respectively), or treatment-as-usual active control. Due to small sample sizes, two classes were combined for randomization purposes; these two were assigned to SP, heretofore referred to as SP-1 (*n* = 6) and SP-2 (*n* = 7), and one class each were assigned to GP (*n* = 12) and control (*n* = 18).

During the 5-week intervention period, participants in the three breathing classes did the 5-min breathing curriculum three times per week. On the 3rd and final class day of each week, they also watched 5-min breath science educational videos and completed the psychometric and physiologic (Supplementary Appendices 3, 4) stress measures before and after that day's breathing practice. Participants in the control classroom completed the psychometric and physiologic stress measures once per week. Final psychometric and physiologic surveys and followup questionnaires were completed during the 1-week follow-up period.

Enrolled students were aware of their assigned curricula only and not whether they were in the treatment or control groups; however, it was not possible to prohibit student interactions that may have led to the unblinding of the treatment assignment. The teacher was not blinded to group assignment.

### Intervention

The slow diaphragmatic breathing curriculum was based on a 6-week, thrice-weekly program that has been found feasible and associated with stress reduction among a small group of high-school student participants (39). The current study evaluated two variations of the slow diaphragmatic breathing curriculum and allowed for practice progressions over the 5 weeks. Students followed 5-min videos for each practice session for the duration of the intervention.

The curriculum incorporates three components that have been shown to significantly reduce psychological and physiological stress responses in both adolescents and other populations (10–24, 28–32): slow breathing; diaphragmatic breathing; and extended exhale breathing. Slow breathing entails breathing at a pace slower than normal breathing. Diaphragmatic breathing focuses on breaths starting from the diaphragm or abdominal areas, with abdominal, then lung, then chest expansion during the inhale and a slow, gradual, full release of air in the reverse direction (chest, lung, diaphragm/abdomen) on the exhale. Extended exhale breathing comprises breathing with the exhalation duration longer than, often twice as long as, the inhalation.

The two variations of slow diaphragmatic extended exhale breathing included in this study differed only in whether participants were taught to pace themselves in the SP classes, or followed a guided pace in the GP class. For both variations, participants were instructed to do the practice while seated comfortably and breathing through the nose. Both variations encouraged or guided gradual slowing of the breathing pace over the 5 weeks; e.g., progressing from a 3-s inhale and 6-s exhale, to 4 and 8, then 5 and 10, respectively. All breathing participants were encouraged to stay with the suggested pace to the extent comfortable, slowing their breathing and extending their exhales without adding any stress or discomfort. They were informed that participants receive benefits of the practice as long as they slow their breathing to slower than their normal breathing, breathe through their noses, breathe from and with the diaphragm, and make the exhales slower than the inhales, even if they do not stay exactly with the guided or suggested pace. Participants were instructed to allow the breath practice to flow comfortably for them.

The curriculum included 5 weekly breath science education videos on the rationale for the practices and how they help with stress reduction. The videos were watched on the assessments day of each week and covered: CO_2_, O_2_, and the feeling of needing to breathe; how breathing regulates the autonomic nervous system; breathing mechanics; and stress-reducing benefits of nasal and slow breathing.

Students in the control class received regular English class instruction during the 5 weeks and completed the assessments once per week.

### Measures

For the study's primary aim, feasibility was based on compliance with the breathing exercises, breath science education videos, and all assessments. Tolerability of the breathing practices and study assessments were measured at followup with surveys asking how easy they were to understand and follow, and how tolerable they were to complete (Supplementary Appendices 5, 6). For the breathing groups, these surveys asked about usefulness and perceived benefit of the curriculum, and included open-ended questions about their experiences. The teacher's tolerability with the intervention, and her evaluation of its benefits and impact on her teaching were measured with a post-curriculum survey (Supplementary Appendix 7).

For the study's secondary aim, preliminary effectiveness of the breathing curriculum was measured with two forms of the STAI psychometric stress survey and a timed-exhale physiological test of stress response (Supplementary Appendices 2–4). STAI-trait was used at baseline and followup to assess participants' generalized, longer-term stress; and STAI-state was used weekly to assess short-term, in-the-moment stress (40). The physiologic test was used at baseline and followup, as well as weekly during the intervention period.

STAI is a self-report questionnaire that has been validated in both adults and adolescents (40, 41) and used in a range of school-based studies (17, 42–45). STAI-Trait is a 20-item scale that evaluates relatively stable aspects of proneness toward anxiety, including general states of calmness, confidence, and security. Final scores from 20 to 80, with higher scores indicating greater levels of stress and anxiety.

STAI-State is also a 20-item scale and evaluates an individual's current state of stress and anxiety, asking about feelings “right now” related to apprehension, tension, nervousness, worry, etc. Because STAI-State was administered weekly, a validated, six-item short version of the survey was used to reduce the time burden and maximize response rates (46) (Supplementary Appendix 4). This shortened version has been shown to have acceptable reliability and validity among various populations, including adolescents (47–51). Final scores range from 6 to 24, with higher scores reflecting higher levels of in-the-moment anxiety.

A timed, maximum-duration exhale test was used to assess participants' physiological tolerance to endogenous CO_2_ as a metric of physiological stress response (52–59). The single-item score reflects the exhale time in seconds, with higher scores indicating greater tolerance to endogenous CO_2_ and physiological stress. Participants were shown a 5-min instructional video in the baseline period and provided written instructions at each administration.

### Analyses

Pearson chi-squared tests were conducted to compare baseline sociodemographic outcomes across classes. To evaluate the feasibility of curriculum implementation, we calculated the number and percent of participants by class complying with the thrice-weekly breathing exercises, the once-weekly breath science videos, and the baseline, weekly, and followup preliminary effectiveness measures (STAI-Trait, STAI-State, and CO_2_TT). To assess curriculum tolerability, we calculated average ease and tolerability ratings across all participants, summarized participants' qualitative assessments by class, and described the teacher's followup survey responses. Average ease and tolerability ratings of the preliminary effectiveness assessments were also calculated and presented across participants.

Means and standard deviations were presented and multivariate analyses of variance were conducted to compare within-class changes in STAI-Trait and CO_2_TT from baseline to followup. Similar analyses were conducted to compare within-class changes in 5-week average STAI-State and CO_2_TT from before to after the breathing exercises. All analyses were conducted using SAS 9.4 for Windows and statistical significance was set at α < 0.05, using two-tailed tests and Tukey adjustments for multiple comparisons. Study treatment group was blinded to the individual who conducted data-cleaning, analyses, and reporting on analyses. Given the feasibility aim of this pilot study and the small sample size, the effectiveness analyses and findings were considered preliminary to help inform future interventions and studies.

## Results

### Population Characteristics

Of the 75 students invited to participate, a total of 43 students (57%) and their parents or guardians provided consent to participate (Figure 1). Baseline demographics were similar among classes (*p* > 0.05), except willingness to disclose smoking/vaping habits (*p* = 0.010; Table 1).

**Table 1:** Participant demographics, by class.

	**Control** *n* = 16^*^	**Self-paced-1** ***n* = 6**	**Self-paced-2** *n* = 6^*^	**Guide-paced** *n* = 10^*^	***p-*Value**
Average age (mean years ± SD)	17.4 (±0.50)	17.5 (±0.55)	17.3 (±0.57)	17.7 (±0.48)	0.37
Gender (% female)	56%	33%	67%	60%	0.66
Ethnicity (% non-Hispanic/White)	94%	100%	100%	100%	0.70
Mother/female guardian education level					0.64
≤high school	0%	0%	17%	10%	
Some college/college graduate	56%	67%	33%	60%	
Graduate degree	44%	33%	50%	30%	
Father/male guardian education level					0.57
≤ high school	6%	0%	0%	0%	
Some college/college graduate	38%	33%	33%	70%	
Graduate degree	56%	67%	67%	30%	
Receiving free/reduced-price lunch	0%	0%	0%	0%	0.70
Diagnosed with asthma	25%	17%	17%	40%	0.67
Among those with asthma
Had asthma attack in prior 12 months	50%	0%	100%	25%	0.66
Taking medication for asthma	25%	0%	0%	25%	0.89
Physically active ≥60 min 4+ days/week	63%	50%	100%	60%	0.35
Do yoga/other meditative practices	31%	33%	33%	40%	0.98
If yes
New practice w/in prior 2 weeks	20%	0%	0%	0%	0.63
Do practice 3+ days/week	20%	100%	0%	100%	0.11
Smoke or vape					0.01
Yes	0%	0%	0%	0%	
Don't know/prefer not to say	0%	33%	0%	0%	

*Group comparisons were conducted with Pearson chi-square tests*.

*SD, standard deviation*.

* *A total of 43 students consented to participate (control, 18; self-paced-1, 6; self-paced-2; 7; guided paced, 12), but not all completed the baseline survey, resulting in the class sample sizes shown here for group comparisons*.

### Feasibility

**Figure 2:**
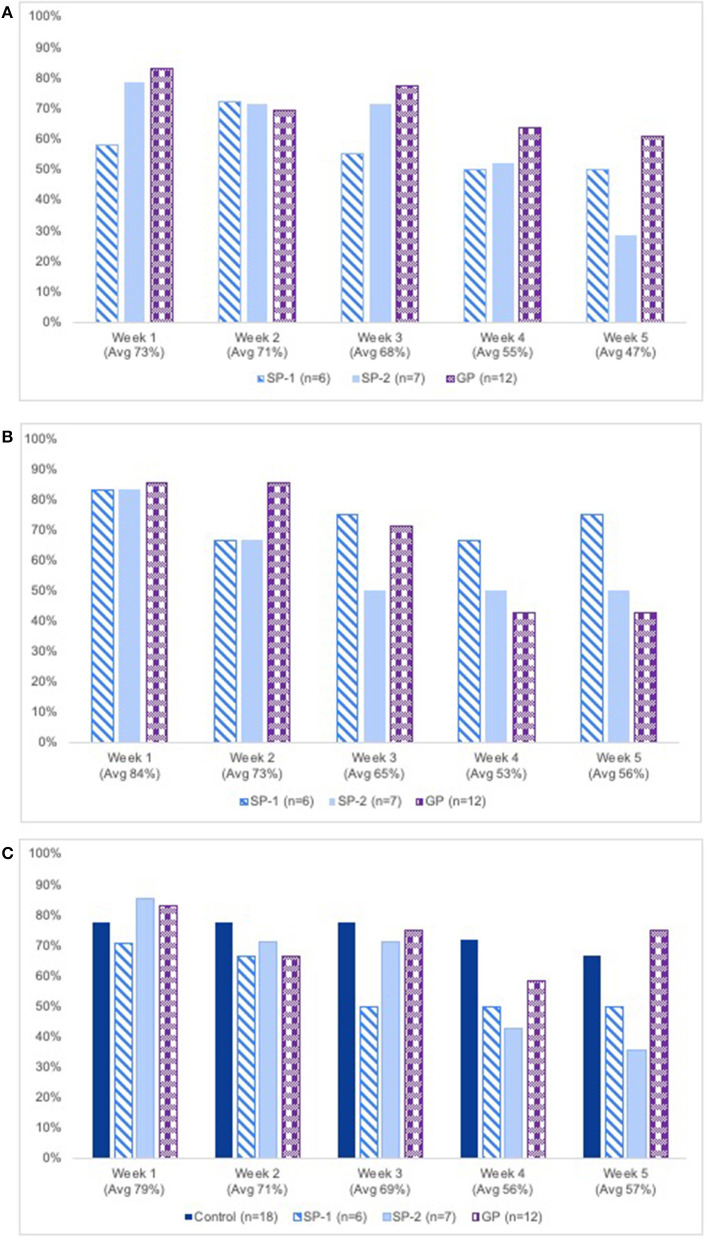
**(A)** Percent of breathing group participants completing breathing exercises, by class & week. GP, guided-pace breathing group. **(B)** Percent of breathing group participants completing breath science education videos, by class & week. GP, guided-pace breathing group. **(C)** Percent of all participants completing STAI-state & CO_2_TT assessments, by class & week. GP, guided-pace breathing group; SP,-1, self-paced group 1; SP,-2, self-paced group 2. CO_2_TT, carbon dioxide tolerance test; STAI, State-Trait Anxiety Inventory.

Compliance with the breathing practices varied from 29 to 83% across classes and weeks and was highest in the GP class in 4 of the 5 weeks (Figure 2A). Averaging across classes, compliance was 73% at week 1 and decreased every week to 47% at week 5. Compliance with the breath science videos ranged from 43 to 86% across weeks and classes (Figure 2B), and that with the weekly STAI-State and CO_2_TT measures varied from 36 to 86% (Figure 2C).

Table 2 shows compliance with baseline and follow-up measures. Baseline compliance was ≥83% for all 4 classes and decreased at followup for all measures.

**Table 2:** Descriptive data for number (%) of participants completing baseline and follow-up measures, by class.

	**Control** ***n* = 18**	**Self-paced-1** ***n* = 6**	**Self-paced-2** ***n* = 7**	**Guide-paced** ***n* = 12**
**STAI-Trait, CO**_**2**_ **TT Assessments**				
Baseline	16 (89%)	5 (83%)	6 (86%)	10 (83%)
Followup	13 (72%)	3 (50%)	2 (29%)	8 (67%)
**Followup ease/tolerability surveys**				
Breath curriculum		3 (50%)	2 (29%)	8 (67%)
Preliminary effectiveness assessments	10 (56%)	2 (33%)	0 (0%)	5 (42%)

*CO_2_TT, carbon dioxide tolerance test; STAI, State-Trait Anxiety Inventory*.

**Figure 3:**
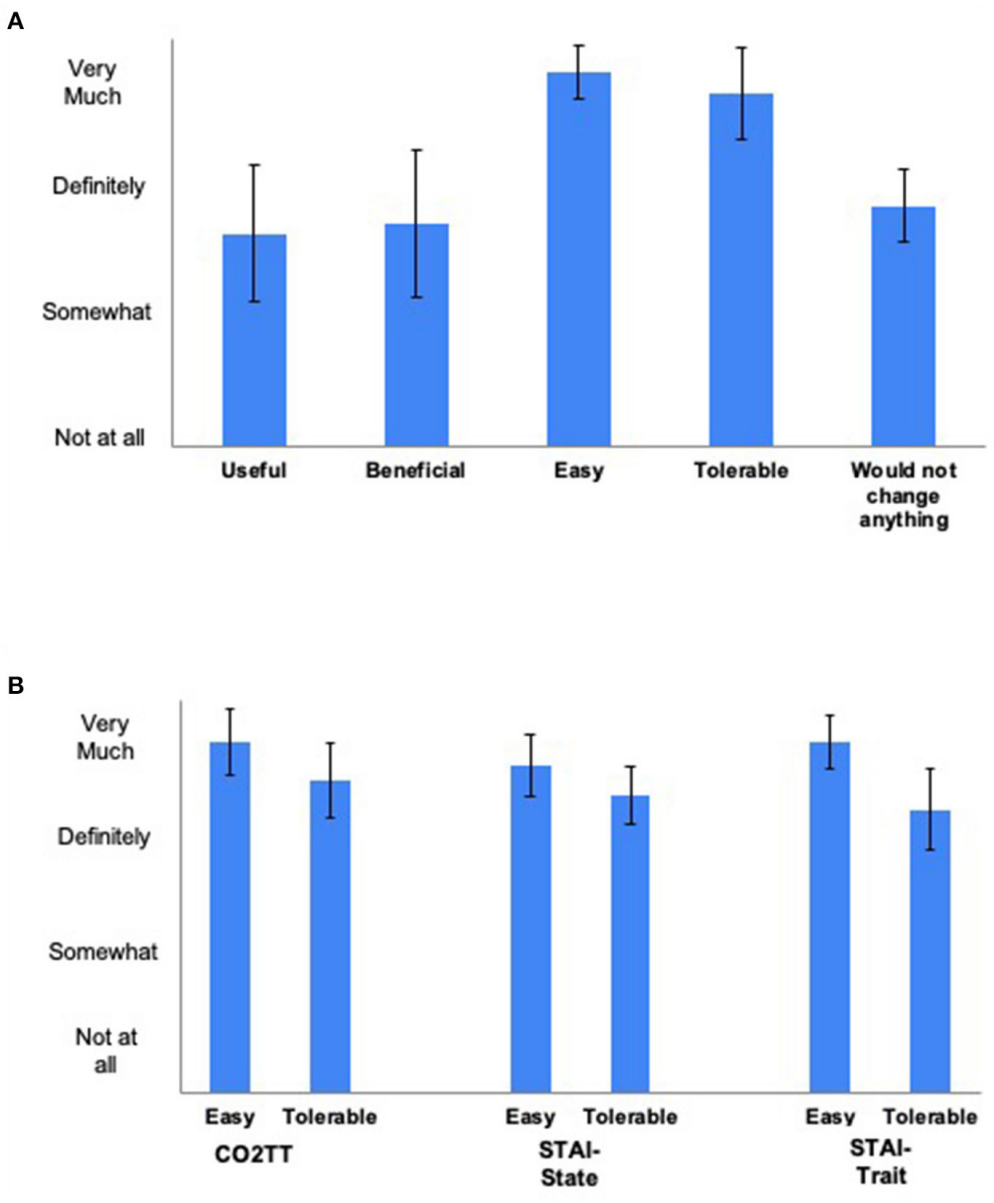
**(A)** Mean (95% confidence intervals) student ratings of the breathing curriculum (*n* =13 student respondents). **(B)** Mean (95% confidence intervals) student ratings of the study assessments (n = 17 student respondents). CO_2_TT, carbon dioxide tolerance test; STAI, State-Trait Anxiety Inventory.

Figures 3A,B show results of the ease and tolerability surveys. On average across classes, the curriculum was rated as somewhat-to-definitely useful and beneficial; and the curriculum and the effectiveness assessments were rated definitely-to-very easy and tolerable. Breathing participants' open-ended comments about the curriculum as a whole are shown in Supplementary Appendix 8. Overall, students reported enjoying the slow diaphragmatic breathing, CO_2_TT, and breath science education videos, although some found some components challenging or time-consuming. Specific positive comments included: students found the breathing exercises to be a nice break and helpful for calming down when stressed; they became more aware of how they breathe throughout the day; their focus increased; they achieved improved breathing during exercise; and they liked seeing improvements in CO_2_TT over time. Specific negative comments included: one student felt discomfort with the extended exhales and another with the CO_2_TT; one student with asthma had some difficulty with the nasal-only and slow-paced breathing; and a few students found various aspects time-consuming. Patterns of positive or negative comments did not vary systematically between classes.

The teacher stated in her survey responses that she felt the breathing exercises were very useful and very beneficial for her students. She reported that implementing the 5-min curriculum somewhat adversely affected her ability to teach the required content for her class; it somewhat benefited her students' abilities to learn the required content for the class; and it did not benefit her ability to teach the class's required content. In response to what she liked best or least about the curriculum, she stated:

“I love this program and the ideas behind it. I believe that helping students self-regulate and focus their attention are extremely important skills for all students. Being in the midst of a global pandemic and having kids at times physically present and at other times on Zoom greatly impacted our participation in the study. A daily breathing practice would be beneficial for all students, and I hope to participate in this program again in the future!”

### Preliminary Effectiveness

Table 3 shows the mean STAI-Trait and CO_2_TT scores at baseline and follow-up by class. There were no significant changes in these measures from before to after the 5-week curriculum implementation. Tables 4A,B show the 5-week mean STAI-State and CO_2_TT scores, respectively, by class from immediately before to after the breathing exercises; no statistically significant effects were present.

**Table 3:** Mean (SD) STAI-Trait and CO_2_TT scores at baseline and follow-up, by class^*^.

**Class**	**Baseline**			**Followup**			***p*** **-values**		
	**STAI-Trait**	**CO_**2**_TT 1**	**CO_**2**_TT 2**	**STAI-Trait**	**CO_**2**_TT 1**	**CO_**2**_TT 2**	**STAI-Trait**	**CO_**2**_TT 1**	**CO_**2**_TT 2**
Cntrl	38.0 (10.6)	17.4 (8.0)	17.4 (7.8)	36.8 (12.4)	17.2 (5.7)	17.1 (5.0)	0.99	1.00	1.00
SP-1	38.9 (9.2)	13.3 (3.9)	12.5 (3.6)	39.3 (8.2)	12.8 (6.3)	12.8 (5.8)	1.00	1.00	1.00
SP-2	38.0 (9.7)	18.9 (5.1)	18.2 (5.0)	35.0 (13.9)	25.5 (6.7)	24.8 (7.6)	0.99	0.66	0.66
GP	38.9 (10.3)	17.3 (7.2)	18.2 (8.4)	37.4 (12.1)	19.5 (7.1)	20.5 (7.6)	0.99	0.94	0.99

*Cntrl, control group; CO_2_TT, carbon dioxide tolerance test; GP, guided-pace group; SD, standard deviation; SP-1, self-paced group 1; SP-2, self-paced group 2; STAI, State-Trait Anxiety Inventory*.

* *Multivariate analyses of variance were conducted to compare within-class changes in STAI-Trait and CO_2_TT from baseline to followup*.

**Table 4A:** 5-week mean (SD) STAI-State scores immediately before vs. immediately after breathing exercises, by class^*^.

**Class**	**Before**		**After**		***p*-values^**‡**^**
	∑n^**^	**Mean (SD)**	∑n^**^	**Mean (SD)**	
**Control**	67	11.51 (4.18)			
**SP-1**	17	11.24 (2.88)	17	9.35 (2.98)	0.69
**SP-2**	22	10.68 (3.36)	21	9.62 (2.75)	0.99
**GP**	43	10.67 (3.27)	43	9.05 (2.89)	0.43

*GP, guided-pace breathing group; SD, standard deviation; SP-1, self-paced group 1; SP-2, self-paced group 2; STAI, State-Trait Anxiety Inventory*.

* *Control class completed STAI-State once per instance; breathing classes completed it before and after that day's breathing exercises*.

** *Total number of participants per class contributing to the 5 weeks' of data*.

‡ *Multivariate analyses of variance were conducted to compare within-class changes in STAI-State from immediately before to immediately after breathing exercises*.

**Table 4B:** 5-week mean (SD) CO_2_TT scores before vs. after breathing exercises, by class^*^.

**Class**	**Before**		**After**		***p-*values^**‡**^**
	∑n^**^	**Mean (SD)**	∑n^**^	**Mean (SD)**	
**Control**	96	18.17 (7.08)	96	18.55 (6.85)	1.00
**SP-1**	26	11.92 (4.63)	25	12.52 (5.11)	1.00
**SP-2**	30	21.62 (5.82)	29	22.41 (5.83)	0.99
**GP**	61	19.25 (8.36)	61	20.23 (8.69)	0.99

*CO_2_TT, carbon dioxide tolerance test; GP, guided-pace breathing group; SD, standard deviation; SP-1, self-paced group 1; SP-2, self-paced group 2*.

* *Control class completed CO_2_TT once before and once after that day's STAI-State; breathing classes completed it before and after that day's breathing exercises*.

** *Total number of participants per class contributing to the 5 weeks' of data*.

‡ *Multivariate analyses of variance were conducted to compare within-class changes in CO_2_TT from immediately before to immediately after breathing exercises*.

## Discussion

We conducted the first pilot study of a slow diaphragmatic breathing curriculum for stress management in a US high school setting. Our results demonstrate that implementing such an intervention is feasible and tolerable in this population, even within the constraints of COVID-era hybrid learning. Consent rates were moderate, with 43 participants out of 75 invited. Compliance with the breath curriculum and the assessments was high in the first 3 weeks, averaging 71−74%, and dropped to 51−54% in the last 2 weeks. Compliance with preliminary effectiveness assessments followed similar patterns: high at ≥83% at baseline and decreasing at follow-up. The breathing exercises were considered very easy and tolerable, and moderately useful and beneficial. In general, students liked the curriculum as-is without recommending changes and reported that they found the practices relaxing, helpful with focus, and calming in times of stress.

For our secondary aim evaluating the curriculum's preliminary effectiveness, the study was able to generate data across a 1.5-week baseline period, 5 weeks of breathing practices, and a 1-week follow-up. Although there were trends toward benefit in STAI-State and CO_2_TT from immediately before to after the breathing practices, it is not surprising that no significant differences were detected, given that the study was not powered for this and the small sample size. Evaluating psychometric and physiologic stress outcomes at different time points was valuable in this study to demonstrate feasibility for future research. Collecting data that provided students with immediate feedback on how the breathing practices affected their stress levels may also have increased the stress-reduction benefits and motivated continued participation. The addition of a low-tech, simple test of physiological stress response such as the CO_2_TT included here further offered participants immediate feedback on the effects of the breathing practices. This was a favorite curriculum component among many students due to the ability to track their progress as well as the self-guided nature of the test.

Results of previous studies indicating effectiveness of slow diaphragmatic breathing even with small samples and similar time frames is encouraging. Chen and colleagues found that anxiety was significantly reduced among 15 high-anxiety adults in Taiwan who practiced diaphragmatic breathing with 12 1:1 guided sessions and twice-daily home practice over 8 weeks (60). Similar evidence exists among other high-anxiety populations (61–63), suggesting that stratifying by baseline anxiety levels may be valuable, as benefits may be more pronounced in those with higher initial stress. Future research with larger sample sizes is warranted to further investigate the benefits of slow diaphragmatic breathing practices on psychometric and physiologic stress outcomes among adolescents.

This curriculum is innovative in providing a fully automated, self-contained, “plug and play” curriculum that requires neither expert trainers skilled in breathing techniques nor changes to existing class curricula; thus, it can be considered both teacher-independent and curriculum supportive. It provides a curriculum that can be delivered consistently across schools and teachers, and in remote or in-person learning environments, increasing the generalizability of its implementation to different settings. The simplicity of the slow breathing practices used in this study render them easy to learn, and the 5-min duration is short enough to encourage implementation even among busy high school students. Prior research has established that the simplest of slow breathing exercises can reduce stress and improve wellbeing, even with just a few minutes of practice (10–16). Students' likelihood of not only adhering to the curriculum but also learning tools for lifelong practice are greater with short, simple practices that once learned, can be done anywhere without the need for audio or video guidance.

### Limitations

The limitations of this study provide valuable insights for the design and implementation of future research. It is hypothesized that the lower-than-expected consent rates, compliance toward study's end, and preliminary effectiveness results are due to two key factors: that curriculum implementation was home-based rather than in-person and in-class; and that it was done in a curriculum-heavy English class. To the first point, the curriculum was designed to be implemented during class in the classroom setting, as a way to both optimize consent and compliance rates, and allow the teacher to provide guidance and feedback for students with the practices. This became infeasible when hybrid learning rendered it challenging at best for the teacher to live-stream the videos to her in-person and at-home students simultaneously, while supervising them and giving them the space to implement the curriculum to fit their needs. This explains why the teacher reported that the curriculum did somewhat adversely affect her ability to teach the required content when she had attempted to implement it during class. To accommodate implementation given these issues, the protocol was changed to instruct participants to implement the breathing exercises and all assessments on their own time at home, after school. This factor alone likely caused our notable compliance declines over time. For example, there was no other classroom-specific or identifiable reason for the 29% followup survey response rate in one class; however, larger sample sizes with in-class implementation would prevent such dramatic percentage drops with even one participants' lack of compliance. For implementation of future programs and research, we recommend that in-class implementation be a priority.

The lack of in-person peer and teacher support may also have contributed to some students' struggles with the extended exhales and CO_2_TT, and their perception that the practices were not extremely beneficial or useful to them. It is important to take such feedback seriously in planning future programs or research. The language in the guided practices should be modified to repeatedly and definitively emphasize—even more than previously—the importance of listening to one's own somatic cues, doing only what feels comfortable, and never allowing the practice to feel stressful in any way. In addition, the teacher should receive more in-depth training on this aspect of the practices and the CO_2_TT so that she/he can better support students throughout the curriculum. Any indications that students are feeling “performance pressure” with the CO_2_TT could be met by the teacher with guidance that increases or decreases in CO_2_TT times are not to be lauded nor chided but rather seen as simple and in fact neutral data points.

Implementing the study in an honors-level English class with its own rigorous curriculum negatively impacted study consent rates, compliance, and perceived benefits of the curriculum. Despite the teacher's enthusiasm and interest in providing this tool for her students, the high academic demands of the class rendered parents and students concerned about spending even 5–10 min of class time on these practices. These concerns were the second contributing factor for changing study implementation to outside of class, on students' own time at home. Implementation of future studies in a class with a wellness-related curriculum—such as health or physical education—is likely to improve all results, from initial study participation to curriculum compliance and effectiveness. Including outcomes such as students' abilities to focus, attendance, or, in a large enough sample, grades may also encourage implementation even in classes with unrelated curricula.

Structural changes, such as more discussions with school personnel and parents to provide more information about the study and increase buy-in, may also help increase consent and compliance rates. These discussions could also be used as a forum to determine the best class type in which to implement the curriculum. Participation incentives could also be offered, such as gift cards for parents or extra credit for students.

The small sample sizes and lower compliance rates toward study's end limit our abilities to draw conclusions about which of the two slow diaphragmatic breathing variations might be more preferable or effective for stress-reduction among high school students. Different approaches may be better for different students, or even for the same student at different times. An ideal curriculum may be one that teaches all students both methods and allows them to choose which to use at any given practice session. Although the two smaller classes did the same self-paced breathing practice, results were presented separately for each class to observe any feasibility and tolerability class differences for our primary aim. Although it is possible that preliminary effectiveness results may have been more powerful with data from these classes combined, analyses for this secondary aim were conducted using the separate class groupings in order to maintain consistency and parsimony within the study design. In future research, exploring various curriculum variations in multiple classrooms and schools will provide deeper insights into both the students' and teachers' experiences.

Table 5 summarizes our recommendations for future implementation.

**Table 5:** Recommendations for future implementation.

**Improve consent rates and compliance by implementing curriculum:**
1. In a class with a health or wellness-directed curriculum; 2. In-person, in the classroom setting; 3. With increased school and parent engagement prior to, during, and following implementation.
**Improve compliance and effectiveness by providing:**
4. Guided instruction that better supports students in adapting the breathing practice pace to meet their bodies' needs, for their comfort and benefit; 5. More teacher training in how to more effectively support and guide students.
**Demonstrate benefits and improve participation by collecting data on:**
6. Short-term stress, such as STAI-State and CO_2_TT, immediately before and after breathing practices to show students the practices' immediate benefits and motivate continued practice; 7. Outcomes such as focus or test-related anxiety to demonstrate academic-related benefits.

*CO_2_TT, carbon dioxide tolerance test; STAI, State-Trait Anxiety Inventory*.

## Conclusions

With stress levels continuing to rise among American adolescents amidst the global pandemic and ever-increasing social and academic pressures, identifying and providing accessible stress management tools to this vulnerable population is critical. Results of this innovative study establish the feasibility of incorporating a slow diaphragmatic breathing curriculum in high school classroom settings and offer valuable insights for increasing participation and effectiveness in future implementation. That the study was completed at all at the peak of the COVID-19 pandemic demonstrates the value of such work to students, families, and teachers alike. For implementation of future programs and research, we recommend that in-class implementation in a health or physical education class be a priority. The continuing investigation of slow diaphragmatic breathing exercises such as those included in this study is warranted to address today's adolescent stress-management crisis and provide youth with tools to last a lifetime.

## Data Availability Statement

The raw data supporting the conclusions of this article will be made available by the authors, without undue reservation.

## Ethics Statement

The studies involving human participants were reviewed and approved by Western IRB - Copernicus Group (WCG IRB). Written informed consent to participate in this study was provided by the participants' legal guardian/next of kin.

## Author Contributions

TB conceived, designed, oversaw, interpreted, and wrote up results of the study and analysis. TB and CS oversaw intervention implementation and data collection. EH helped conceive the study design, conceived and developed the study curriculum, and recruited the study site. TB, EH, BM, and RW developed the study interventions. TB and KL developed analytic design and oversaw data collection. AV developed data collection tools and contributed to data collection and recording. NA and AC contributed to data interpretation and manuscript writing. KL analyzed study data. All authors reviewed and approved the final manuscript.

## Funding

This study received funding from individual charitable donations to the Health and Human Performance Foundation, a registered 501(c)(3) non-profit organization #83-2727288. The donors were not involved in the study design, collection, analysis, interpretation of data, and the writing of this article or the decision to submit it for publication.

## Conflict of Interest

The authors declare that the research was conducted in the absence of any commercial or financial relationships that could be construed as a potential conflict of interest.

## Publisher's Note

All claims expressed in this article are solely those of the authors and do not necessarily represent those of their affiliated organizations, or those of the publisher, the editors and the reviewers. Any product that may be evaluated in this article, or claim that may be made by its manufacturer, is not guaranteed or endorsed by the publisher.

## Abbreviations

CO_2_: carbon dioxide
CO_2_TT: carbon dioxide tolerance test
GP: guide-paced (breathing)
SD: standard deviation
SP: self-paced (breathing)
STAI: State Trait Anxiety Inventory
min: minute
s: second.
