# Supplementary material for: Slow-Breathing Curriculum for Stress Reduction in High School Students: Lessons Learned From a Feasibility Pilot
Source: Front Rehabil Sci. 2022 Jul 1;3:864079. doi: 10.3389/fresc.2022.864079 (PMC9397716; doi:10.3389/fresc.2022.864079)
Supplement: Supplementary file 1 [file Table_1.docx]

# **Supplementary Appendix 1. Baseline Survey**

Please answer the following questions.

1. Your age (years): ___________ or prefer not to say
2. The gender you identify with (circle one): male female nonbinary prefer not to say
3. Race/ethnicity (circle one):

A. African-American

B. Non-Hispanic/White

C. Latino or Hispanic

D. Asian

E. Native American

F. Native Hawaiian or Pacific Islander

G. Two or More

H. Other/Unknown

I. Prefer not to say

1. Mother/female guardian’s highest education level:
   1. <= high school
   2. Some college/college graduate
   3. Graduate degree
   4. Don’t know or prefer not to say
   5. Not applicable
2. Father/male guardian’s highest education level:
   1. <= high school
   2. Some college/college graduate
   3. Graduate degree
   4. Don’t know or prefer not to say
   5. Not applicable
3. Are you receiving free or reduced-price lunch this year? yes/no/prefer not to say
4. Have you ever been told by a doctor that you have asthma? yes/no/prefer not to say

If yes:

1. have you had an asthma attack in the past 12 months? yes/no/prefer not to say
2. Are you currently taking a daily medication for your asthma that was prescribed or given to you by your doctor? yes/no/prefer not to say
3. During a typical week, on how many days are you physically active for at least 60 minutes total per day? Do not include PE.

________ days or don’t know or prefer not to say

1. Do you do yoga, meditation, or breathing practices (either on your own, with others, using an app, or other)?

If yes:

- 1. Is this a new practice in the past 2 weeks? yes/no/prefer not to say
  2. How many days per week do you practice for at least 5 minutes? [<2, 3-5, 6+, don’t know, prefer not to say]

1. Do you smoke? yes/no/prefer not to say
2. If yes, how many cigarettes do you smoke per day? [<2, 3-10, 11+, don’t know, prefer not to say]
