# Supplementary material for: Slow-Breathing Curriculum for Stress Reduction in High School Students: Lessons Learned From a Feasibility Pilot
Source: Front Rehabil Sci. 2022 Jul 1;3:864079. doi: 10.3389/fresc.2022.864079 (PMC9397716; doi:10.3389/fresc.2022.864079)
Supplement: Supplementary file 2 [file Table_2.docx]

# **Supplementary Appendix 2. STAI-Trait Survey and Scoring**

A number of statements that people have used to describe themselves are given below. Read each statement and then circle the appropriate number to the right of the statement to indicate how you *generally* feel. There are no right or wrong answers. Do not spend too much time on any one statement but give the answer that seems to describe how you generally feel.

|  |  | **Almost never** | **Sometimes** | **Often** | **Almost always** |
| --- | --- | --- | --- | --- | --- |
| 1 | I feel pleasant | 1 | 2 | 3 | 4 |
| 2 | I feel nervous and restless | 1 | 2 | 3 | 4 |
| 3 | I feel satisfied with myself | 1 | 2 | 3 | 4 |
| 4 | I wish I could be as happy as others seem to be | 1 | 2 | 3 | 4 |
| 5 | I feel like a failure | 1 | 2 | 3 | 4 |
| 6 | I feel rested | 1 | 2 | 3 | 4 |
| 7 | I am “calm, cool, and collected” | 1 | 2 | 3 | 4 |
| 8 | I feel that difficulties are piling up so that I cannot overcome them | 1 | 2 | 3 | 4 |
| 9 | I worry too much over something that really doesn’t matter | 1 | 2 | 3 | 4 |
| 10 | I am happy | 1 | 2 | 3 | 4 |
| 11 | I have disturbing thoughts | 1 | 2 | 3 | 4 |
| 12 | I lack self-confidence | 1 | 2 | 3 | 4 |
| 13 | I feel secure | 1 | 2 | 3 | 4 |
| 14 | I make decisions easily | 1 | 2 | 3 | 4 |
| 15 | I feel inadequate | 1 | 2 | 3 | 4 |
| 16 | I am content | 1 | 2 | 3 | 4 |
| 17 | Some unimportant thought runs through my mind and bothers me | 1 | 2 | 3 | 4 |
| 18 | I take disappointments so keenly that I can’t put them out of my mind | 1 | 2 | 3 | 4 |
| 19 | I am a steady person | 1 | 2 | 3 | 4 |
| 20 | I get in a state of tension or turmoil as I think over my recent concerns and interests | 1 | 2 | 3 | 4 |

**Scoring**

Total the scoring weights shown for each response category below. For example, for question #1, if the respondent marked 3 for “often,” then the scoring weight for that response would be 2. The final, single-value STAI-trait score is the total of all scoring weights for the 20 items.

|  | **Almost never** | **Sometimes** | **Often** | **Almost always** |
| --- | --- | --- | --- | --- |
| **1** | 4 | 3 | 2 | 1 |
| **2** | 1 | 2 | 3 | 4 |
| **3** | 4 | 3 | 2 | 1 |
| **4** | 1 | 2 | 3 | 4 |
| **5** | 1 | 2 | 3 | 4 |
| **6** | 4 | 3 | 2 | 1 |
| **7** | 4 | 3 | 2 | 1 |
| **8** | 1 | 2 | 3 | 4 |
| **9** | 1 | 2 | 3 | 4 |
| **10** | 4 | 3 | 2 | 1 |
| **11** | 1 | 2 | 3 | 4 |
| **12** | 1 | 2 | 3 | 4 |
| **13** | 4 | 3 | 2 | 1 |
| **14** | 4 | 3 | 2 | 1 |
| **15** | 1 | 2 | 3 | 4 |
| **16** | 4 | 3 | 2 | 1 |
| **17** | 1 | 2 | 3 | 4 |
| **18** | 1 | 2 | 3 | 4 |
| **19** | 4 | 3 | 2 | 1 |
| **20** | 1 | 2 | 3 | 4 |
