# Supplementary material for: Slow-Breathing Curriculum for Stress Reduction in High School Students: Lessons Learned From a Feasibility Pilot
Source: Front Rehabil Sci. 2022 Jul 1;3:864079. doi: 10.3389/fresc.2022.864079 (PMC9397716; doi:10.3389/fresc.2022.864079)
Supplement: Supplementary file 3 [file Table_3.docx]

# **Supplementary Appendix 3. CO2 Tolerance Test Instructions**

The carbon dioxide tolerance test (CO2TT) is a simple test of the human body’s

response to blood CO2 levels. The CO2TT is very easy and both time and cost effective, as it requires very little time and only a stopwatch to complete.

Be sure when performing the CO2TT to:

● Perform the test at approximately the same time each day (as other circadian

factors may affect the outcome);

● Sit down a few minutes before beginning the test and during this time, refrain from:

○ Eating or drinking anything besides water

○ Digital screen use

○ Any conversations or activity that causes excitability

● Have a stopwatch or timer available

All breathing for the CO2TT is done **through the nose only**. Your mouth should not

open for any reason during the entire test. If it does, the test is invalid and needs to be

performed again.

With mouth closed, follow these simple steps to perform the test:

1. Take three normal, complete breaths, where the inhale is approximately 2

seconds in duration and the exhale is approximately 2 seconds in duration. There

should be no purposeful pauses between the two phases of the breath cycle.

2. Inhale a fourth time and fill your lungs as thoroughly as possible, to maximal

capacity.

3. Start your timer and exhale continuously as slowly as possible, continuing until you either:

● Run out of air

● Pause, stop, or otherwise interrupt the continuous exhale

● Swallow

● Inhale or decide to breathe in for any other reason than running out of air

● Panic

4. Stop the timer and take a relaxed breath in as soon as any of these occur.

5. Record your time:

● This maximal exhale is timed with a stopwatch. The time begins as soon

as you begin to exhale and ends when your continuous exhale is stopped

or interrupted for any of the above reasons.

● The time of your continuous exhale is your CO2TT Score. Be sure to

record this score along with time, date, and location of your test.
