# Supplementary material for: Slow-Breathing Curriculum for Stress Reduction in High School Students: Lessons Learned From a Feasibility Pilot
Source: Front Rehabil Sci. 2022 Jul 1;3:864079. doi: 10.3389/fresc.2022.864079 (PMC9397716; doi:10.3389/fresc.2022.864079)
Supplement: Supplementary file 4 [file Table_4.docx]

# **Supplementary Appendix 4. STAI-State (Short version)**

A number of statements which people have used to describe themselves are given below. Read each statement and then circle the most appropriate number to the right of the statement to indicate how you feel right now, at this moment. There are no right or wrong answers. Do not spend too much time on any one statement but give the answer which seems to describe your present feelings best.

|  | **Not at all** | **Somewhat** | **Moderately** | **Very Much** |
| --- | --- | --- | --- | --- |
| **1. I feel calm** | 1 | 2 | 3 | 4 |
| **2. I am tense** | 1 | 2 | 3 | 4 |
| **3. I feel upset** | 1 | 2 | 3 | 4 |
| **4. I am relaxed** | 1 | 2 | 3 | 4 |
| **5. I feel content** | 1 | 2 | 3 | 4 |
| **6. I am worried** | 1 | 2 | 3 | 4 |

**Scoring:**

The scoring weights shown for each response category below are totaled for one final score ranging from 6 to 24. For example, for question #1, if the respondent marked 3 for “moderately,” then the scoring weight for that response would be 2. The final, single-value STAI-state (short) score is the total of all scoring weights for the 6 items.

| **Response #** | **Not at all** | **Somewhat** | **Moderately** | **Very Much** |
| --- | --- | --- | --- | --- |
| **1.** | 4 | 3 | 2 | 1 |
| **2.** | 1 | 2 | 3 | 4 |
| **3.** | 1 | 2 | 3 | 4 |
| **4.** | 4 | 3 | 2 | 1 |
| **5.** | 4 | 3 | 2 | 1 |
| **6.** | 1 | 2 | 3 | 4 |
