# Supplementary material for: Slow-Breathing Curriculum for Stress Reduction in High School Students: Lessons Learned From a Feasibility Pilot
Source: Front Rehabil Sci. 2022 Jul 1;3:864079. doi: 10.3389/fresc.2022.864079 (PMC9397716; doi:10.3389/fresc.2022.864079)
Supplement: Supplementary file 5 [file Table_5.docx]

# **Supplementary Appendix 5. Participant Breathing Curriculum Survey**

Please think about this curriculum as a whole over the last 5 weeks:

1. In general, how **useful** was the 5-minute breathing practice curriculum?

0 Not useful 1 Somewhat useful 2 Useful 3 very useful

1. Did you experience any **benefit from participating** in this 5-minute breathing practice curriculum?

0 No benefit 1 Benefited somewhat 2 Benefited 3 Benefited greatly

1. Did you find the 5-minute guided breath practices **easy** to understand and follow?

0 Not easy 1 Somewhat easy 2 Easy 3 very easy

1. Did you find the 5-minute guided breath practices **tolerable**?

0 Very tolerable 1 Mostly tolerable 2 Somewhat tolerable 3 Not tolerable

1. Is there anything you would change about the 5-minute guided breath practices curriculum that would make it more beneficial, easier for you to complete, and/or more tolerable?

0 No 1 Yes

Open-ended questions:

1. In general, did you like the breathing curriculum? Why or why not?
2. Which part(s) did you like best about the curriculum? For example, did you prefer the breathing practices, the CO2TT, the breath science videos, or the stress surveys? Why?
3. Which part(s) did you like least? Why?
4. Please add any other comments about the curriculum or assessments.
