# Supplementary material for: Slow-Breathing Curriculum for Stress Reduction in High School Students: Lessons Learned From a Feasibility Pilot
Source: Front Rehabil Sci. 2022 Jul 1;3:864079. doi: 10.3389/fresc.2022.864079 (PMC9397716; doi:10.3389/fresc.2022.864079)
Supplement: Supplementary file 6 [file Table_6.docx]

# **Supplementary Appendix 6. Participants Assessments Experience Survey**

In general, during the past 5 weeks of the curriculum:

1. Was the CO2TT **easy** to understand and follow?

0 Very easy 1 Easy 2 Somewhat easy 3 Not easy

1. Did you find the CO2TT **tolerable** to do?

0 Very tolerable 1 Mostly tolerable 2 Somewhat tolerable 3 Not tolerable

1. Was the 6-item STAI-State assessment **easy** to understand and follow?

0 Very easy 1 Easy 2 Somewhat easy 3 Not easy

1. Did you find the 6-item STAI-State assessment **tolerable** to complete?

0 Very tolerable 1 Mostly tolerable 2 Somewhat tolerable 3 Not tolerable

1. Was the 20-item STAI-Trait assessment (completed during pre- and post-curriculum periods) **easy** to understand and follow?

0 Very easy 1 Easy 2 Somewhat easy 3 Not easy

1. Did you find the 20-item STAI-Trait assessment **tolerable** to complete?

0 Very tolerable 1 Mostly tolerable 2 Somewhat tolerable 3 Not tolerable
