# Supplementary material for: Slow-Breathing Curriculum for Stress Reduction in High School Students: Lessons Learned From a Feasibility Pilot
Source: Front Rehabil Sci. 2022 Jul 1;3:864079. doi: 10.3389/fresc.2022.864079 (PMC9397716; doi:10.3389/fresc.2022.864079)
Supplement: Supplementary file 7 [file Table_7.docx]

# **Supplementary Appendix 7. Teacher Curriculum Experience Survey**

**Please think about this program as a whole over the last 6 weeks.**

1. In general, how **useful** do you feel this curriculum was for your students?

0 Very useful 1 Useful 2 Somewhat useful 3 Not useful

1. Do you perceive that your students experienced any **benefit from participating** in this curriculum?

0 Benefited greatly 1 Benefited 2 Benefited somewhat 3 No benefit

1. Did implementing the 5-minute breathing curriculum:
2. **adversely impact your ability to teach** the required educational content for your class?

0 No adverse impact 1 Some adverse impact 2 Adverse impact 3 Large adverse impact

1. **benefit** **your students’ abilities** to learn the required educational content for your class?

0 Benefited greatly 1 Benefited 2 Benefited somewhat 3 No benefit

1. **benefit** **your ability** to **teach** the required educational content for your class?

0 Benefited greatly 1 Benefited 2 Benefited somewhat 3 No benefit

**Please add any other comments about the curriculum, assessments or their implementation in your classroom**, including what you liked best/least and what worked best/least:

**_________________________________________________________________________**

**_________________________________________________________________________**

**_________________________________________________________________________**

**_________________________________________________________________________**
