# Supplementary material for: Slow-Breathing Curriculum for Stress Reduction in High School Students: Lessons Learned From a Feasibility Pilot
Source: Front Rehabil Sci. 2022 Jul 1;3:864079. doi: 10.3389/fresc.2022.864079 (PMC9397716; doi:10.3389/fresc.2022.864079)
Supplement: Supplementary file 8 [file Table_8.docx]

## Supplementary Appendix 8. Breathing participants’ qualitative comments at follow-up regarding breathing curriculum, by class

**Did you like the breathing curriculum? Why / why not?**

*Self-paced 1*

- I did not like it very much as I found the extended exhales to make me a little panicky
- No because how I felt after

*Self-paced 2*

- In general, I liked it, however, it seemed to take a lot of time and it could have been faster.
- I liked it! I found it very relaxing and beneficial in terms of making me focused.

*Guide-paced*

- Yes! It was super helpful to get relaxed and helped me be more mindful of my breath throughout the day.
- Yeah it allowed me to just reflect
- Yes, it really fascinates me how much my C02TT changes each day and I really enjoy watching that fluctuate.
- In general, I liked the breathing curriculum it was nice to do when I was super stressed out because it would calm me down.
- Yes, I felt like I learned things and how to be more mindful of how I breathe.
- Yes, I saw some benefits with my focus and my breathing while exercising.
- I kind of liked it.
- Yes, it was a nice break to clear my headspace before class

**Which part did you like best? Why?**

*Self-paced 1*

- I liked the CO2TT surveys.
- Breathing practices
- It was ok, it just didn't do anything for me.

*Self-paced 2*

- I liked the CO2TT the best.
- I liked the 5-minute breathing practice videos.
- [Breath science] videos

*Guide-paced*

- I honestly love the 5-minute breath practice because it was a set time for me to really focus on my breath and de-stressing. I also liked the CO2TT test after the 5 minutes because I was able to do longer and that was cool to see.
- I liked the CO2TT because I saw my progress
- The C02TT is my favorite part because it is self-guided.
- I liked the breathing practices and the science videos the most. I think it helped me be more mindful of my breathing throughout the day, and reduced my stress levels.
- The science videos and the breathing practices were my favorite.
- I liked the breath science videos because they helped my understanding of why breathing is important.
- I liked the stress surveys.
- Breath science videos as they were easy to follow and beneficial

**Which part(s) did you like least? Why?**

*Self-paced 1*

- I did not like the extended breathing practices
- Surveys
- [Breath science] videos

*Self-paced 2*

- I disliked the stress surveys because they were time-consuming and very repetitive.
- The breath science videos, they took a lot of time

*Guide-paced*

- Sometimes it was hard for me to follow the breath science videos. I thought they were really cool and definitely think they were helpful and should stay but they were sometimes hard for me to follow.
- The surveys they were repetitive
- The videos honestly are just difficult to engage in after a while. They become very monotonous.
- There wasn't anything I didn't like per se, but sometimes the stress surveys were tedious.
- The CO2TT test because I feel like I didn't have much improvement and I was upset about that.
- The stress surveys because I didn't see the benefit of doing them.
- It was okay, I have asthma so at times it was kind of tricky to perform the exercise. I treat my asthma; however I did not particularly enjoy the part of the practice where it was only through your nose.
- Not sure

**Other comments:**

*Self-paced 1*

- Stress surveys: same questions over and over

*Self-paced 2* [no other comments]

*Guide-paced*

- Thank you for letting us be a part of this!
- Overall, it was a well-designed curriculum and experiment. I think the results might be a bit skewed because of all the confounding variables of being at home. I also liked the breathing and have been using it when I get stressed out.
- It was good I just panicked easily on the CO2TT
